# Super-twisting ADRC for maximum power point tracking control of photovoltaic power generation system based on non-linear extended state observer

**DOI:** 10.1016/j.heliyon.2024.e36428

**Published:** 2024-08-17

**Authors:** Amir Hossein Raouf, Fatemeh Sadat Yazdiniya, Gholam Reza Ansarifar

**Affiliations:** a Department of Nuclear Engineering, Faculty of Physics, University of Isfahan, Isfahan, Iran; b Department of Mechanical Engineering, Faculty of Engineering, University of Isfahan, Isfahan, Iran

**Keywords:** Active disturbance rejection control (ADRC), Super-twisting sliding mode control (ST-SMC), Extended state observer (ESO), PV array, Maximum power point tracking (MPPT)

## Abstract

This paper proposed a new method for maximum power point tracking in photovoltaic power generation systems by combining super-twisting sliding mode control and active disturbance rejection method. An incremental guidance method is used to find the point of maximum power. The non-linear extended state observer is applied to estimate the unmodeled dynamics and external disturbance. The ADRC based on a super-twisting sliding mode is designed to bring the state variables to the desired state. In the next step, the stability of NESO and ADRC are theoretically proved. Finally, the simulation results have been compared with the results of the PI controller, classical sliding mode control, and terminal sliding mode control (TSMC) presented in other articles. The results show the effectiveness and superiority of the proposed method.

Also, to check the performance of the proposal method in real-time, real-time results have been compared with non-real-time results. The results obtained from the real-time and non-real-time simulations exhibited a minimal difference. This fact indicates the high accuracy of the modeling and simulations performed. Indeed, the mathematical models and non-real-time simulations have been able to accurately mimic the actual behavior of the photovoltaic system under various operating conditions.

## Highlights

• This paper proposed a new method for maximum power point tracking in photovoltaic power generation systems.
• The non-linear extended state observer is applied to estimate the un-modeled dynamics and external disturbance.
• The stability of NESO and ADRC are theoretically proved.
• The simulation results have been compared with the results of PI controller and TSMC.
• The results show the effectiveness and superiority of the proposed method.

**Table:** Nomenclature

$I_{ph}$	Photocurrent of the PV panel (A)
$I_{o}$	Reverse saturation current of the PV panel (A)
$I_{or}$	Reverse saturation current at the PV panel reference temperature (A)
$I_{scr}$	Short-circuit cell current at the PV panel reference temperature (A)
$N_{P}$	Number of cells connected in parallel
$N_{S}$	Number of cells connected in series
A	Ideality factor of the P–N junction
K	Boltzmann's constant (J/K)
$K_{i}$	Short-circuit current temperature coefficient (A/K)
T	Temperature (K)
$T_{r}$	Reference temperature (K)
q	Electronic charge (C)
$E_{g}$	Band-gap energy of the semiconductor (ev)
E	Solar radiation (mW/cm^2^)
d	Duty ratio of the control input signal
$C_{1}$	Input capacitance (F)
$C_{2}$	Output capacitance (F)
R	Load resistance (Ω)
$R_{C}$	Internal resistance on the capacitance (Ω)
L	Inductor inductance (H)
$V_{D}$	Forward voltage of the diode (V)
$i_{L}$	Inductor current (A)
$V_{pv}$	PV panel output voltage (V)
$V_{C2}$	The voltage of the capacitor $C_{2}$ (V)
$V_{ref}$	Reference maximum power PV voltage (V)
$I_{pv}$	PV panel output current (A)
$P_{pv}$	PV panel power (W)

## 1 Introduction

Energy supply has always been one of the important challenges in the development of countries. The increase in population and the need for economic growth are two big drivers for the growth of energy demand. Due to the concerns caused by the depletion of fossil fuels and their environmental problems, the use of renewable energy, especially solar panels, has expanded [1,2]. Solar batteries or solar cells are electronic devices that convert light or photons directly into electric current or voltage using the photovoltaic phenomenon. One of the advantages of solar cells is their free and non-polluting fuel, and its disadvantages are the high initial cost and low efficiency. Due to environmental changes, including temperature, radiation, etc., PV panels have a non-linear nature, which reduces their efficiency. Therefore, achieving the maximum power point under these conditions is very important [3]. To get the most power from the solar cell, a specific current must be drawn from the cell. The technique of tracking the maximum power point indicates the same issue in such a way that the maximum power is received [4].

Maximum power point tracking is an electrical system that allows PV modules to achieve their maximum power. There is a maximum point in the power-voltage characteristic curve, which is called MPP. The purpose of MPPT is to apply the appropriate voltage to both ends of the solar array to receive the most current from the array. To extract more power from the PV array, various methods including a look-up table, and curve fitting have been applied to maximum power point tracking [5,6]. Maximum power tracking methods are divided into two categories: traditional methods and optimization methods. Traditional methods include perturbation and observation methods, incremental guidance methods, and hill-climbing methods. Optimization methods include fuzzy logic [7], neural networks, and other optimization methods [8]. These methods are easy to implement and cost-effective. One of the disadvantages of this method is the fluctuation around the maximum power point [9]. The perturbation and observation (P & O) method has been significantly used in PV panels due to the simplicity of the control system and the small number of measurement parameters [10]. This method has disadvantages such as dependence on constant weather conditions or prolonged changes [11]. To solve this problem, the method of perturbation and adaptive observation with variable steps is presented, in which step changes are adjusted according to the change of some parameters such as power changes [12,13].

To overcome the losses of chaos method and observation of the incremental conduction method is used [14,15]. In the incremental conduction method, the output voltage or current of the PV panels is adjusted in a way that the current ratio to the output voltage PV is equal to the incremental conduction $\frac{dI}{dV}$. When any deviation is observed, the ratio of current to voltage changes so that this ratio is equal to $\frac{dI}{dV}$ , because at this point the maximum power is obtained [16]. This method works well when environmental conditions change and its fluctuation around the working point is insignificant. In the methods of fuzzy control and neural networks, the maximum power point tracking is done by considering different atmospheric conditions and PV panel parameters, but complex calculations and the need to use a microcontroller are seen in these methods and they are difficult to implement.

Many maximum power point tracking methods are stable only near the maximum power point and unstable elsewhere. To implement easily and ensure stability, the maximum power point voltage is developed based on the two-stage MPPT control scheme. The task of the first step is to determine the desired voltage of the maximum power point of the PV panel and the task of the second step is to bring the PV panel voltage to the desired voltage. Searching for the reference voltage and bringing the PV panel voltage to the reference voltage is repeated until the system reaches the maximum power point.

Reaching maximum power is dependent on the tracking controller performance in the second step. Different approaches are proposed for voltage regulation including the traditional sliding mode controller [17,18], fuzzy logic MPPT method, and PI control [19], MPC method [20], and terminal sliding mode control [21].

The good functioning of a control system depends on the stability, robustness, and accuracy of the system in tracking the path. These criteria have a direct relationship with the accuracy of the dynamic model and the adjustment of the control parameters. These cases have led to the use of the active disturbance rejection control method. This method is resistant to the uncertainties of the model and can remove the disturbance instantly without affecting the output. For the first time, ADRC was proposed by Han [22]. This proposal by Gao [23] was introduced in the English language. ADRC consists of two important parts: An Extended state observer to estimate unmodeled dynamics, uncertainties, and external disturbance as total disturbance and a suitable controller to reject disturbance. Applying non-linear controllers to PV panels provides a suitable response due to their non-linear nature. One of the non-linear robust controllers is the sliding mode control. The sliding mode controller is a non-linear controller with features such as accuracy, robustness, ease of adjustment, and implementation. In the sliding mode control, the state trajectories are directed to a certain surface in the state space, which is called the sliding surface [24]. So far, this control method has been widely used for photovoltaic panels [[25], [26], [27]]. One of the disadvantages of sliding mode control is the chattering phenomenon, which occurs on the sliding surface. To solve this problem, high-order sliding mode controllers can be used. In Ref. [28], a nonlinear high-gain observer-based second-order sliding mode (SOSM) control strategy for grid-connected neutral-point-clamped converters is proposed. The NHGO technique addresses the challenge of measurement noise in observer-based controllers by using a time-varying gain, which is high during transients and low during steady-state conditions. This adaptive gain minimizes the negative impact of measurement noise while maintaining the observer's effectiveness. In Ref. [29] presents a novel control strategy for neutral-point-clamped (NPC) power converters, combining the strengths of proportional-integral control and super-twisting algorithm through a varying exponent gain approach. The proposed method allows the controller to switch smoothly between PI and STA modes, providing smooth control input and robustness against disturbances. A higher-order sliding-mode observer is integrated into the control scheme. This observer compensates for external disturbances, ensuring accurate estimation and improved disturbance rejection capabilities. The results show fast dynamic response, strong disturbance rejection, and minimized chattering. In this paper, a super-twisting sliding mode control is used in the ADRC structure which, in addition to eliminating chattering, has a better convergence time than the traditional model. In Ref. [30] a second-order sliding mode controller is used to eliminate the chattering phenomenon to control the converter. In Ref. [31] super-twisting algorithm is designed for a photovoltaic system. Active disturbance removal control has been widely used on PV panel systems so far [[32], [33], [34], [35]], but its combination with super-twisting sliding mode control is rare. In Ref. [36] for air management in polymer electrolyte membrane fuel cell (PEMFC) systems, a control method utilizing a linear extended state observer (LESO) and sliding mode technique is presented. The primary objective is to prevent oxygen starvation during sudden load changes, which can damage the polymer membranes. The controller comprises two loops: an outer loop that estimates the oxygen excess ratio and generates a reference compressor flow rate, and an inner loop that regulates the actual compressor flow rate. The experimental results show the controller's ability to respond rapidly to load changes, with less settling time. A key advantage of this method is that it achieves performance comparable to state feedback control without requiring direct measurement of the oxygen excess ratio. This makes it a practical and effective solution for preventing oxygen starvation in PEMFC systems, particularly during sudden load variations. In Ref. [37] introduces an innovative adaptive disturbance observer-based fixed-time backstepping control algorithm designed to handle uncertain robotic systems. An adaptive sliding mode disturbance observer (ASMDO) is designed to compensate for disturbances such as model uncertainty and friction. Additionally, an adaptive fixed-time auxiliary system is developed to address the problem of actuator saturation in robotic systems. This combination enables the proposed control algorithm to achieve high-precision tracking control within a fixed time, regardless of the initial conditions. The proposed approach is particularly effective in practical scenarios requiring robust performance under various disturbances.

The contribution of this paper is the super-twisting ADRC-based MPPT design for PV power generation systems. The general system consists of a PV panel connected to a boost converter. The reference voltage is obtained from the incremental conduction method and the system reaches the maximum power point based on the active disturbance rejection method. The purpose of this article is to improve the tracking speed of the maximum power point and reduce fluctuations under external and internal disturbances in PV panels. The results are compared with the terminal sliding mode controller (TSMC) presented in Ref. [21], the classical sliding mode control presented in Ref. [18], and the PI controller. Finally, the results of real-time simulation and non-real-time simulation are compared.

This article is compiled in six parts. In the second part, the modeling and system equations and in the third part, the design of the active disturbance rejection controller is presented. In the fourth section, the stability of the NESO and controller is proved. In the fifth part, the simulation results are presented, and finally, in the sixth part, the conclusion is presented.

## 2 Photovoltaic system modeling

### 2.1 Model and characteristics of the photovoltaic panels

**Fig. 1:**
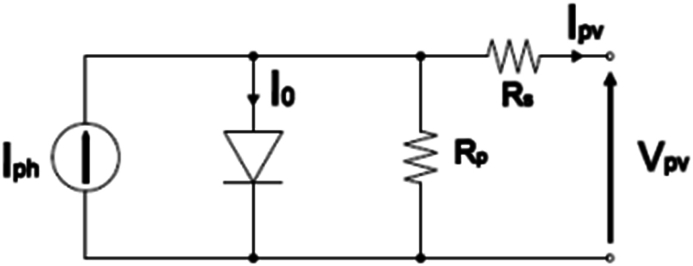
PV panel equivalent circuit model.

The most famous and best model to model the photovoltaic cell is a parallel current source with a diode which is paralleled and in series with a resistor and is shown in Fig. 1. The equation of the output current is as follows [38]:

(1) $$I_{pv}=N_{p}I_{ph}-N_{p}I_{o}\left(\exp\left(\frac{qV_{pv}}{N_{s}AKT}\right)-1\right)$$

where $I_{pv}$ and $V_{pv}$ are the output current and the output voltage of the PV panel, respectively. The value of the other parameters is presented in Table 1. The saturation current of the PV panel is expressed as follows:

(2) $$I_{o}=I_{Or}{\left(\frac{T}{T_{r}}\right)}^{3}\;\exp\;\left(\frac{qE_{g}}{KT}\left(\frac{1}{T_{r}}-\frac{1}{T}\right)\right)$$

**Table 1:** Parameter of the PV array.

Symbol	Parameter	Value
$P_{\max}$	Maximum output Power	200W±10%
$i_{\max}$	Maximum Current	7.61A
$V_{oc}$	Open circuit voltage	32.9V
$i_{scr}$	Short circuit current	8.21A

The reverse saturation current $I_{Or}$ at the PV panel reference temperature $T_{r}$ is defined by:

(3) $$I_{or}=\exp\;\left(\frac{I_{scr}}{\exp\;\left(\frac{qV_{oc}}{N_{s}AKT}\right)-1}\right)$$

The photocurrent of the PV panel $I_{ph}$ is dependent on the temperature $T_{r}$ and radiation. Obtained from the following equation:

(4) $$I_{ph}=\left(I_{scr}+K_{i}\left(T-T_{r}\right)\right)\frac{E}{1000}$$

the PV module power is given by:

(5) $$\begin{array}{c} P_{pv} \end{array}=V_{pv}I_{pv}=N_{p}I_{ph}V_{pv}-N_{p}I_{o}V_{pv}\left(\exp\left(\frac{qV_{PV}}{N_{S}AKT}\right)-1\right)$$

### 2.2 Boost converter modeling

**Fig. 2:**
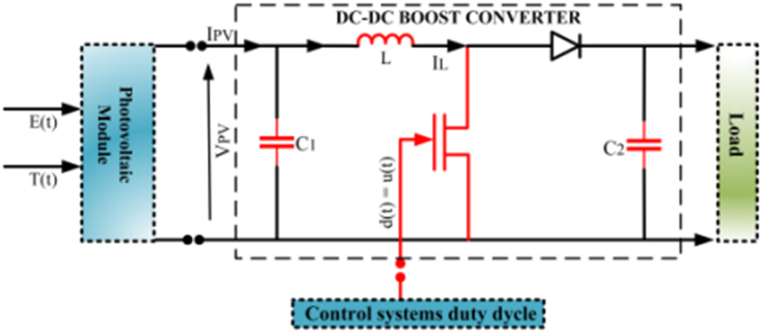
Internal structure of DC-DC converter in photovoltaic system [39].

In the form of photovoltaic systems, a step-up DC-DC converter is used to achieve the maximum power point. Which consists of electronic elements including an input capacitor, inductor, diode, load resistor, MOSFET transistor, and output capacitor [39,40]. The internal structure of the converter is presented in Fig. 2. The dynamic equations related to the above DC-DC converter are presented in Equation (7).

(6) $$\begin{array}{c} {\dot{V}}_{pv}=\frac{-1}{C_{1}}I_{L}+\frac{I_{pv}}{C_{1}}\\ {\dot{I}}_{L}=\frac{1}{L}V_{pv}-\frac{R_{C}\left(1-d\right)}{L\left(1+\frac{R_{C}}{R}\right)}I_{L}+\frac{1-d}{L}\left(\frac{R_{c}}{R+R_{C}}-1\right)V_{C2}-\frac{V_{D}\left(1-d\right)}{L}\\ {\dot{V}}_{C2}=\frac{1-d}{C_{2}\left(1+\frac{R_{C}}{R}\right)}I_{L}-\frac{1}{C_{2}\left(R+R_{C}\right)}V_{C2} \end{array}$$

Where $V_{pv}$, $V_{C2}$, and $I_{L}$ are the PV module output voltage of the capacitance $C_{1}$, the voltage of the capacitance $C_{2}$, and the current on the inductance L, respectively. Also, $R_{C}$ is the internal resistance on the capacitance $C_{2}$, R is the load resistance, $V_{D}$ is the forward voltage of the diode, and d(t) represents the duty cycle signal applied to the DC-DC converter. The numerical values of these constants are presented in Table 2.

**Table 2:** Parameters of converter, and maximum power point tracking.

Parameter	Value
K	$1.3805\times 10^{-23}\;J/K$
$K_{i}$	$4.79\times 10^{-3}\;A/C$
q	$1.6\times 10^{-19}\;C$
A	1.8
$R_{L}$	0.15Ω
$R_{C}$	39.6Ω
$C_{1}$	1000μF
$C_{2}$	1000μF
R	25Ω
$V_{D}$	0.82V
ΔV(MPPT)	0.0005
$V_{ref}\left(MPPT\right)$	26.3V

By defining the vector of state variables to the form $\left(t\right)={\left[\begin{array}{ccc} x_{1}\left(t\right) & x_{2}\left(t\right) & x_{3}\left(t\right) \end{array}\right]}^{T}={\left[\begin{array}{ccc} V_{pv} & i_{L} & V_{C2} \end{array}\right]}^{T}$ and control input u(t) as d(t), the dynamic equation (6) are converted into the state space model:

(7) $$\left\{ \begin{array}{c} {\dot{x}}_{1}=\frac{1}{C_{1}}\left({-x}_{2}+i_{pv}\right)\\ {\dot{x}}_{2}=f_{1}\left(x\right)+g_{1}\left(x\right)d\left(t\right)\\ {\dot{x}}_{3}=f_{2}\left(x\right)+g_{2}\left(x\right)d\left(t\right) \end{array}\right.$$

Where:

(8) $$f_{1}\left(x\right)=\frac{x_{1}}{L}-\frac{R_{C}}{L\left(1+\frac{R_{C}}{R}\right)}x_{2}+\frac{1}{L}\left(\frac{R_{c}}{R+R_{C}}-1\right)x_{3}-\frac{V_{D}}{L}$$

(9) $$g_{1}\left(x\right)=-\frac{R_{C}}{L\left(1+\frac{R_{C}}{R}\right)}x_{2}-\frac{1}{L}\left(\frac{R_{c}}{R+R_{C}}-1\right)x_{3}+\frac{V_{D}}{L}$$

(10) $$f_{2}\left(x\right)=\frac{1}{C_{2}\left(1+\frac{R_{c}}{R}\right)}x_{2}-1\frac{1}{C_{2}\left(R+R_{C}\right)}x_{3}$$

(11) $$g_{2}\left(x\right)=-\frac{1}{C_{2}\left(1+\frac{R_{C}}{R}\right)}x_{2}$$

## 3 Control methodology

To receive the maximum power from the PV panel under internal and external disturbance conditions, the maximum power reference voltage is generated by the MPPT algorithm.

### 3.1 MPPT algorithm

To get the point where the output power is maximum, the derivative of power concerning voltage is defined as follows [5]:

(12) $$\frac{dP_{pv}}{dV_{pv}}=I_{pv}+V_{pv}\frac{{dI}_{pv}}{{dV}_{pv}}$$

According to Equation (12), the production power will be maximum when the above expression is equal to zero. That's mean: dPpvdVpv=0.

According to the above explanation, the $V_{ref}$ voltage update law will be as follows:

(13) $$\left\{ \begin{array}{c} V_{ref}\left(k\right)=V_{ref}\left(k-1\right)+\Delta V,\frac{d_{Ipv}}{{dV}_{pv}}>-\frac{I_{pv}}{V_{pv}}\\ V_{ref}\left(k\right)=V_{ref}\left(k-1\right)-\Delta V,\frac{d_{Ipv}}{{dV}_{pv}}<-\frac{I_{pv}}{V_{pv}} \end{array}\right.$$

Where $V_{ref}\left(k\right)$ is the reference MPV at k th step, ΔV it is an update parameter that can be determined experimentally. From several iterations, the maximum power generation condition with dPpvdVpv=0 is obtained. Therefore, the problem changes to controlling the voltage of the PV array to follow the MPV reference voltage, which is $V_{ref}$.

**Fig. 3:**
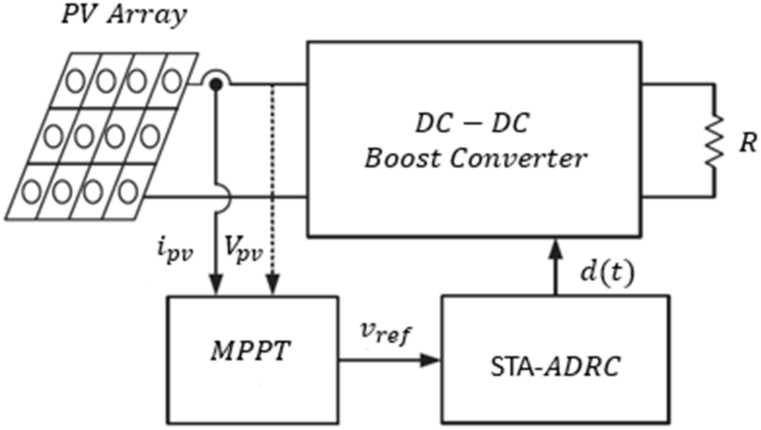
MPPT structure.

In the following, as the main goal of control, the applied input d(t) to the photovoltaic control system should be designed so that after the finite time, variable $V_{pv}$ reaches exactly the selected reference $V_{ref}$. Therefore, a resistant controller with a short convergence time is needed to be able to bring $V_{pv}$ to $V_{ref}$ and be resistant to disturbances. MPPT structure is presented in Fig. 3.

### 3.2 Non-linear extended state observer

The second-order NESO is designed as:

(14) $$\left\{ \begin{array}{c} e=z_{1}-x_{1}\\ {\dot{z}}_{1}=z_{2}-l_{1}e\\ {\dot{z}}_{2}=z_{3}+b_{0}u\left(t\right)-l_{2}e\\ {\dot{z}}_{3}=-l_{3}fal\left(e,\sigma ,\delta \right) \end{array}\right.$$

Where $z_{1}$ is an estimation of $x_{1}$, $z_{2}$ is an estimation of the derivate of $x_{1}$ $,z_{3}$ is the extended state for estimation of generalized disturbance ($f_{1}\left(x\right)$), $e=z_{1}-y$ is an estimation error, $l_{i},i=1,2,3$ is observer gains, and $b_{0}$ is the control input gain. By presenting a suitable proposal to reduce the parameters that need to be adjusted, Zhao [41] puts all the poles of the observer in one point and expresses the benefits of the observer in terms of a function of one parameter. For this purpose, the characteristic Equation of system (14) is set equal to the desired characteristic equation, all of whose roots are at $-\omega_{0}\text{.}$ It results in the characteristic polynomial of (14) to be

(15) $$s^{3}+l_{l}s^{2}+l_{2}s+l_{3}={\left(s+\omega_{0}\right)}^{3}$$

Where $\omega_{0}$ is considered as the bandwidth of the observer and $l_{1}=3\omega_{0},l_{2}=3\omega_{0}^{2},l_{3}=\omega_{0}^{3}$.

The advantages of this approach are twofold: first simplified tuning: only one parameter needs to be adjusted, reducing the complexity of the design process. Second predictable behavior: the single-point pole placement leads to a more consistent and predictable observer response. The selection of $\omega_{0}$ is crucial and should be based on the system dynamics and performance requirements. A higher $\omega_{0}$ generally leads to faster estimation but may increase sensitivity to noise, while a lower $\omega_{0}$ results in slower but smoother estimation.

The nonlinear function fal(.) is introduced to enhance the observer's performance in the presence of noise and disturbances. This function is defined as:

(16) $$fal\left(e,\sigma ,\delta \right)=\left\{ \begin{array}{cc} \frac{e}{\delta^{1-\sigma }} & \left|e\right|\leq \delta \\ {\left|e\right|}^{\sigma }sign\left(e\right) & \left|e\right|\geq \delta \end{array}\right.$$

Where 0<σ<1, δ>0.

### 3.3 Super-twisting sliding mode control

Robust control approaches are the best type of controller for systems under severe disturbances. Among all the non-linear robust control methods, the sliding mode control method has been the most prominent in recent decades due to its high robustness and simplicity of design. These types of controllers, using a control law with high switching speed, set the system state variables at a certain level called the sliding level. Contrary to the classical sliding mode control, the second-order sliding control based on the super-twisting algorithm does not need to determine the sign of the derivative of the sliding surface $\dot{s}$ and feedback on the high-order derivatives of the state variables to determine the sliding surface. The super-twisting algorithm provides a control with better performance against uncertainties and external disturbances without the need to calculate the sliding surface and by using the previous measurement information. Also, the chattering caused by the classic sliding mode is significantly reduced.

By leveraging the robustness and fast response characteristics of super-twisting sliding mode control along with the disturbance rejection capabilities of ADRC, the method effectively regulates the system to achieve the desired operating state. This integration ensures that the state variables remain within the desired boundary, enhancing the stability and performance of the system.

If $x_{1}$ is the real state and $x_{1}^{d}=V_{ref}$ is the desired trajectory, tracking error is defined as $\varepsilon_{1}=x_{1}-x_{1}^{d}$, ${\dot{\varepsilon }}_{1}=\frac{1}{C_{1}}\left(-x_{2}+I_{pv}\right)-{\dot{x}}_{1}^{d}\text{.}$

If $x_{2}$ considered as virtual control input, The auxiliary error is written as:

$$\varepsilon_{2}=x_{2}-x_{2}^{d},x_{2}^{d}=I_{pv}-C_{1}{\dot{x}}_{1}^{d}\text{.}$$

So; the new derivative of the errors is defined as ${\dot{\varepsilon }}_{1}=\frac{-\varepsilon_{2}}{C_{1}}$, ${\dot{\varepsilon }}_{2}=f_{1}\left(x\right)+g_{1}\left(x\right)d\left(t\right)-{\dot{x}}_{2}^{d}$.

Where:

$${\dot{x}}_{2}^{d}={\dot{I\;}}_{pv}-C_{1}{\ddot{x}}_{1}^{d}\text{.}$$

In the first step to design the super-twisting sliding mode controller, the sliding surface is defined:

(17) $$s={\left(\lambda +\frac{d}{dt}\right)}^{n-1}\varepsilon_{1}=\varepsilon_{2}+\lambda \varepsilon_{1}$$

Where λ is a positive definite gain and n is the system order and according to Equation (7) is 2.

The ST-SMC law is defined as follows [42]:

(18) $$d_{eq}\left(t\right)={\dot{x}}_{2}^{d}+\frac{\lambda \varepsilon_{2}}{C_{1}}-k_{1}{\left|s\right|}^{\frac{1}{2}}sign\left(s\right)-k_{2}\int_{0}^{t}sign\left(s\left(\tau \right)\right)d\tau$$

$k_{1},k_{2}$ are positive definite gains of the ST-SMC. where sign(s) represents the sign function which is defined as follows:

(19) $$sign\left(s\right)=\left\{ \begin{array}{cc} 1, & if\;s>0\\ -1, & if\;s<0\\ 0, & otherwise \end{array}\right.$$

The efficacy of the super-twisting sliding mode controller is intrinsically linked to its control gain parameters. Increasing λ enhances the convergence rate, albeit at the expense of increased control effort and maximum overshoot. The gain $k_{1}$ primarily determines the system's response speed. An increment in $k_{1}$ yields improved controller performance but concurrently exacerbates chattering and maximum overshoot. Conversely, the gain $k_{2}$ affects the steady-state error. A reduction in $k_{2}$ mitigates steady-state error, though this is accompanied by an intensification of chattering. Furthermore, the ratio between $k_{1}$ and $k_{2}$ plays a crucial role in system stability and performance. This ratio must be chosen to satisfy the Lyapunov stability condition.

The controller design process necessitates finding an optimal ratio between $k_{1}$ and $k_{2}$ that maximizes overall system efficiency. The super-twisting sliding mode controller's performance is highly robust against disturbances and uncertainties. However, fine-tuning of parameters is essential to achieve desired performance.

The selection of key parameters focuses on ensuring the negative definiteness of the Lyapunov function's derivative, which guarantees the asymptotic stability of the closed-loop system. Control gains, especially $k_{1}$, are adjusted to minimize the duration needed for the system state to reach the sliding surface. To reduce overshoot, which is the maximum deviation from the desired setpoint, adjustments are made, often involving a trade-off with convergence time, as faster convergence may lead to increased overshoot. The gains, particularly $k_{2}$, are fine-tuned to reduce the steady-state error, which is the difference between the desired and actual output as time approaches infinity. Additionally, care is taken to minimize chattering, the high-frequency oscillation that can occur in sliding mode control systems.

### 3.4 Tracking differentiator (TD)

TD is a filter. It can produce the original signal with better quality and its derivative without noise [43]. In this paper, to reduce overshoot and undershoot and increase the speed of convergence, the optimal TD presented in Ref. [44] is applied:

(20) $$\left\{ \begin{array}{c} {\dot{v}}_{1}=v_{2}+hv_{2}\\ {\dot{v}}_{2}=v_{2}+hu,\left|u\right|\leq r \end{array}\right.$$

(21) $$u=fhan\left(v_{1}-v,v_{2},r,h,a\right)$$

Where v is the desired signal, $v_{1}$ is the main quality signal, $v_{2}$ is the derivative of $v_{1}$, fhan is the ideal function defined in Ref. [44]. The r,h, and a used for regulation the speed of convergence and accuracy of the optimal tracking differentiator.

### 3.5 ADRC structure

**Fig. 4:**
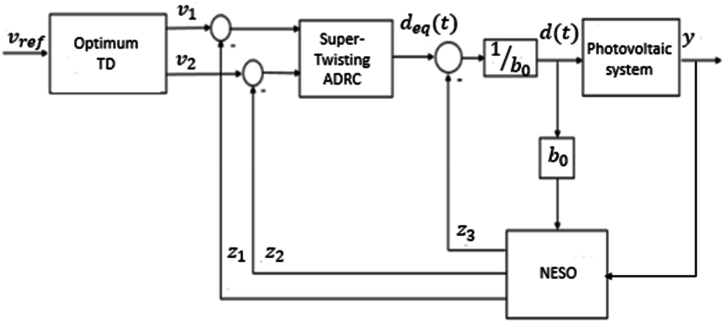
Super twisting-ADRC structure.

Active disturbance rejection-based ST-SMC is presented in Fig. 4.

Using equation (11), (19) control law is modified as follows:

(22) $$d\left(t\right)=\frac{1}{b_{0}}\left(d_{eq}\left(t\right)-z_{3}\right)$$

Indeed, the super-twisting algorithm maintains a high tracking performance of the controller and allows the control input to eliminate chattering. To estimate the uncertainties and compensate for disturbances, a nonlinear disturbance observer is presented to improve the disturbance rejection. A controller using STR-ADRC achieves high precision, and the simulation results demonstrate the validity of the proposed control approach.

## 4 Stability analysis of closed loop dynamics

### 4.1 Finite-time convergence of ESO

The non-linear extended state observer estimation error dynamic can be written as:

(23) $$\left\{ \begin{array}{c} {\dot{e}}_{1}=e_{2}-l_{1}e_{1}\\ {\dot{e}}_{2}=e_{3}-l_{2}e_{1}\\ {\dot{e}}_{3}=-l_{3}fal\left(e_{1}\right)-H\left(t\right) \end{array}\right.$$

Let $\sigma_{i}=\frac{e_{i}}{\omega_{0}^{i-1}}$ , i=1,2,3. Then equation (23) can be written as:

(24) $$\dot{\sigma }=\omega_{0}A\sigma +B\frac{H\left(t\right)}{\omega_{0}}$$

where $A=\left[\begin{array}{ccc} -a_{1} & 1 & 0\\ -a_{2} & 0 & 1\\ -a_{3} & 0 & 0 \end{array}\right],B=\left[\begin{array}{c} 0\\ 0\\ 1 \end{array}\right]$,

Theorem 1 Assuming H(t) is bounded. There exists a positive constant $p_{i},i=1,2,3$ and a finite time T>0 such that $\left|e_{i}\right|\leq p,i=1,2,3,\forall t\geq T,\omega_{0}>0$.

Proof. Solving (24), it can be obtained:

(25) $$\sigma \left(t\right)=e^{\omega_{0}At}\sigma \left(0\right)+\int_{0}^{t}e^{\omega_{0}A\left(t-\tau \right)}B\frac{H\left(\tau \right)}{\omega_{0}^{2}}d\tau$$

Let

(26) $$p=\int_{0}^{t}e^{\omega_{0}A\left(t-\tau \right)}B\frac{H\left(\tau \right)}{\omega_{0}^{2}}d\tau$$

Since |H(t)|≤D and ${\left(e^{\omega_{0}A\left(t-\tau \right)}B\right)}_{i}>0,i=1,2,3$ then

$$P_{i}\left(t\right)=\int_{0}^{t}{\left(e^{\omega_{0}A\left(t-\tau \right)}B\right)}_{i}\frac{H\left(\tau \right)}{\omega_{0}^{2}}d\tau$$

$$=\frac{1}{{\omega_{0}}^{2}}\int_{0}^{t}{\left(e^{\omega_{0}A\left(t-\tau \right)}B\right)}_{i}H\left(\tau \right)d\tau$$

$$\leq \frac{D}{\omega_{0}^{3}}\int_{0}^{t}{\left(e^{\omega_{0}A\left(t-\tau \right)}\right)}_{i}d\tau$$

$$=\frac{D}{{\omega_{0}}^{2}}{\left(e^{\omega_{0}At}\right)}_{i}\int_{0}^{t}{\left(e^{{-\omega }_{0}A\tau }B\right)}_{i}d\tau$$

$$=\frac{D}{{\omega_{0}}^{2}}{\left(e^{\omega_{0}At}\right)}_{i}{\left(\frac{1}{-\omega_{0}A}\right)}_{i}{\left(e^{-\omega_{0}AT}B\right)}_{i}{|}_{0}^{t}$$

$$=\frac{D}{{\omega_{0}}^{3}}{\left(e^{\omega_{0}At}\right)}_{i}{\left(\frac{1}{-\omega_{0}A}\right)}_{i}{\left(e^{-\omega_{0}At}B-B\right)}_{i}$$

$$=\frac{D}{\omega_{0}^{3}}{\left(\frac{1}{-A}\right)}_{i}{\left(B-e^{-\omega_{0}At}B\right)}_{i}$$

(27) $$=\frac{D}{\omega_{0}^{3}}\left[{\left(-A^{-1}B\right)}_{i}+{\left(A^{-1}e^{\omega_{0}At}B\right)}_{i}\right],i=1,2,3$$

Hence

(28) $$\begin{array}{c} \left|p_{i}\left(t\right)\right|\leq \frac{D}{\omega_{0}^{3}}\left[{\left|\left(-A^{-1}B\right)\right|}_{i}+{\left|\left(A^{-1}e^{\omega_{0}At}B\right)\right|}_{i}\right],i=1,2,3\\ \text{Since}\\ \begin{array}{c} A^{-1}=\left[\begin{array}{ccc} 0 & 0 & -a_{1}\\ 1 & 0 & -a_{2}\\ 0 & 1 & -a_{3} \end{array}\right]\\ \text{Then} \end{array} \end{array}$$

(29) $$\left|{\left(A^{-1}B\right)}_{i}\right|=\left\{ \begin{array}{cc} 1, & i=1,\\ 2, & i=2,3. \end{array}\right.$$

Since A is a Hurwitz matrix, there exists a finite time T>0, that for all t≥T,

(30) $$\left\{ \begin{array}{c} \left|{\left(e^{\omega_{0}At}\right)}_{i,j}\right|\leq \frac{1}{\omega_{0}^{2}},i,j=1,2\\ \left|{\left(e^{\omega_{0}At}\right)}_{i}\right|\leq \frac{1}{\omega_{0}^{2}},i=1,2 \end{array}\right.$$

From equation (25), it can be acquired

(31) $$\left|\sigma_{i}\right|\leq \left|{\left[e^{\omega_{0}At}\sigma \left(0\right)\right]}_{i}\right|+\left|p_{i}\right|$$

Let $e_{s}\left(0\right)=e_{1}\left(0\right)+\frac{\left|e_{2}\left(0\right)\right|}{\omega_{0}}+\frac{\left|e_{3}\left(0\right)\right|}{\omega_{0}^{2}}$ according to $\sigma_{i}\left(t\right)=\frac{e_{i}\left(t\right)}{\omega_{0}^{i-1}}$, then

(32) $$\left|e_{i}\left(t\right)\right|\leq \left|\frac{e_{sum}\left(0\right)}{\omega_{0}^{3}}\right|+\frac{2\sigma }{\omega_{0}^{4-i}}+\frac{4\sigma }{\omega_{0}^{7-i}}=p_{i},i=1,2,3$$

The finite time convergence is proved. According to (16), the estimation errors $e_{1}$ and $e_{2}$ depend on the ESO gains $l_{1},l_{2},l_{3},\sigma$ and δ. By tuning gains correctly, the estimation error will be small enough, that is, the estimated states will reach the actual states of the system. Also, the gain of estimation of disturbances and uncertainties is chosen big enough to be able to estimate them. $l_{1},l_{2},l_{3}>0$ is the basic criterion for parameter tune.

### 4.2 Controller stability analysis

Consider the general super-twisting sliding mode control law as follows:

(33) $$d\left(t\right)={g_{1}\left(x\right)}^{-1}\left({\dot{x}}_{2}^{d}+\frac{\lambda \varepsilon_{2}}{C_{1}}-k_{1}{\left|s\right|}^{\frac{1}{2}}sign\left(s\right)-k_{2}\int_{0}^{t}sign\left(s\left(\tau \right)\right)d\tau -f_{1}\left(x\right)\right)$$

Assumption 1 All the state variables of the PV panel are measurable or observable.

Assumption 2 The reference voltage and its first and second derivatives are bounded.

Assumption 3 $f_{1}\left(x\right)$ is bounded.

**Proof.** The Lyapunov function for sliding surface is considered as follows:

For equation (17), the Lyapunov function is constructed:

(34) $$V=\frac{1}{2}s^{2}$$

Theorem 2 According to Lyapunov’s theory, the derivative of V should be $\dot{V}\leq 0$

(35) $$\dot{V}=s\dot{s}=s\left(\varepsilon_{2}+\lambda \varepsilon_{1}\right)$$

$$=s\left(g_{1}\left(x\right)d\left(t\right)+f_{1}\left(x\right)-{\dot{x}}_{2}^{d}-\frac{\lambda \varepsilon_{2}}{C_{1}}\right)$$

If $z_{3}=\hat{f}\left(x\right)$ and considering $\frac{g_{1}\left(x\right)\;}{b_{0}}∼1$ and considering equation (33):

$$\dot{V}=s\left({\dot{x}}_{2}^{d}+\frac{\lambda {\hat{\varepsilon }}_{2}}{C_{1}}-k_{1}{\left|\hat{s}\right|}^{\frac{1}{2}}sign\left(\hat{s}\right)-k_{2}\int_{0}^{t}sign\left(\hat{s}\left(\tau \right)\right)d\tau -\hat{f}\left(x\right)+f_{1}\left(x\right)-{\dot{x}}_{2}^{d}-\frac{\lambda \varepsilon_{2}}{C_{1}}\right)$$

(36) $$=s\left(-k_{1}{\left|\hat{s}\right|}^{\frac{1}{2}}sign\left(\hat{s}\right)-k_{2}\int_{0}^{t}sign\left(\hat{s}\left(\tau \right)\right)d\tau +\tilde{f}\left(x\right)-\frac{\lambda {\tilde{\varepsilon }}_{2}}{C_{1}}\right)$$

(37) $$=s\left(-k_{1}{\left|-\lambda {\tilde{\varepsilon }}_{1}-{\tilde{\varepsilon }}_{2}+s\right|}^{\frac{1}{2}}sign\left(-\lambda {\tilde{\varepsilon }}_{1}-{\tilde{\varepsilon }}_{2}+s\right)\;-k_{2}\int_{0}^{t}sign\left(-\lambda {\tilde{\varepsilon }}_{1}-{\tilde{\varepsilon }}_{2}+s\left(\tau \right)\right)d\tau +\tilde{f}\left(x\right)-\frac{\lambda {\tilde{\varepsilon }}_{2}}{C_{1}}\right)$$

Where $\tilde{e},\dot{\tilde{e}}$ are non-linear extended state errors. Considering Theorem 1, since the NESO can make the errors converge to zero, Equation (37) can be expressed as follows:

(38) $$\dot{V}\approx s(-k_{1}\left({\left|s\right|}^{\frac{1}{2}}sign\left(s\right)-k_{2}\int_{0}^{t}sign\left(s\left(\tau \right)\right)d\tau \right)$$

Therefore, if only $k_{1},k_{2}>0$, then $\dot{V}\leq 0$, the closed-loop system is stable.

## 5 Simulation results

**Fig. 5:**
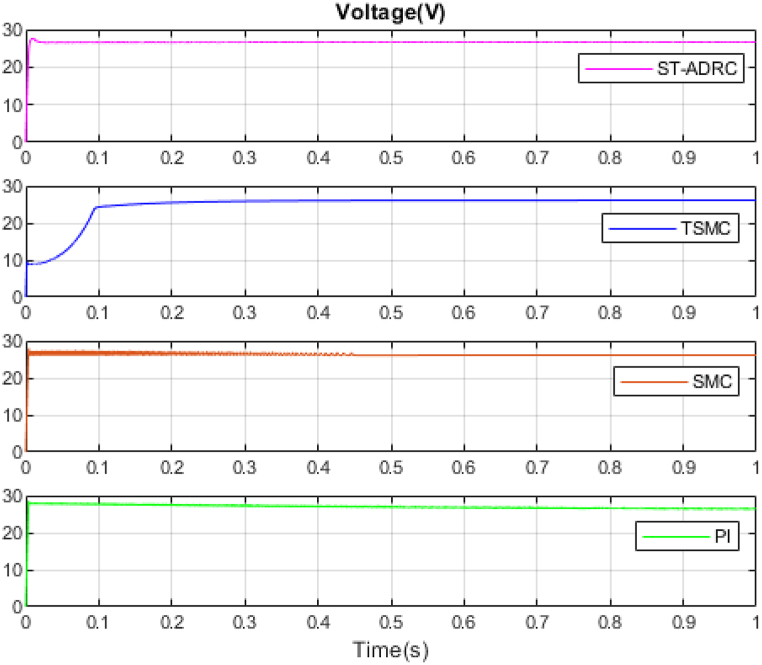
PV panel voltage (V).

**Fig. 6:**
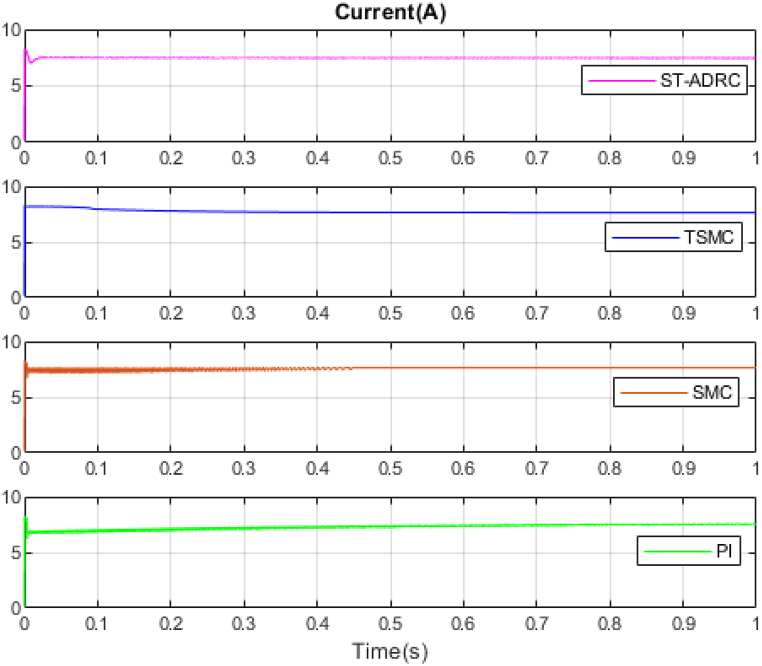
PV panel current (A).

**Fig. 7:**
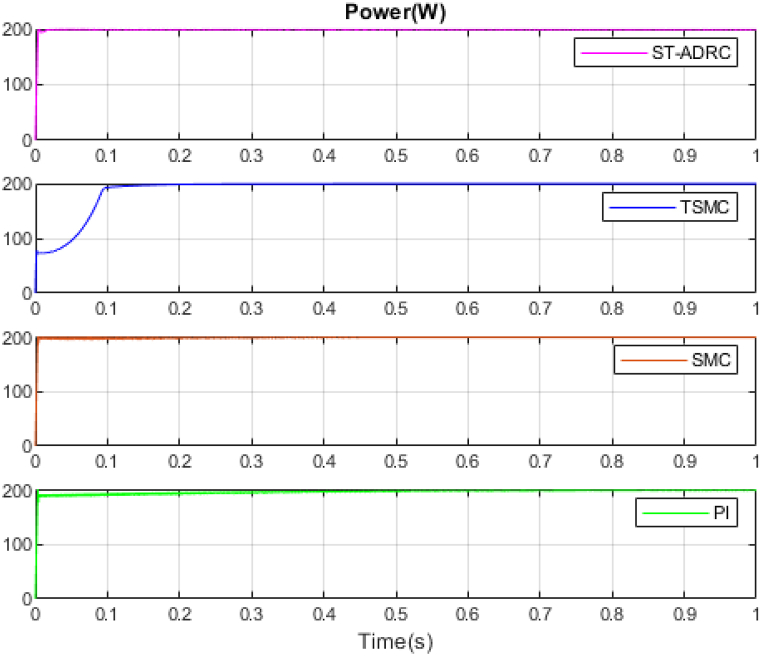
PV panel power (W).

**Fig. 8:**
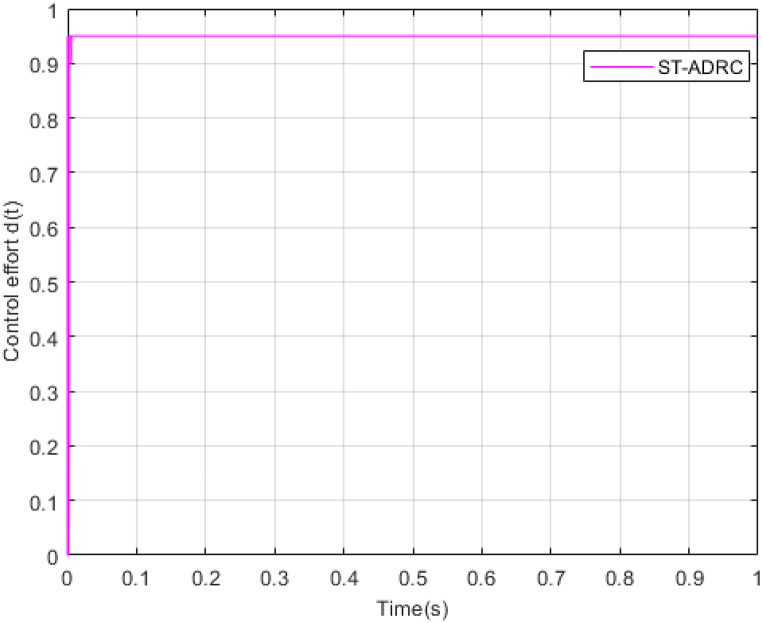
PV Control effort d(t).

**Fig. 9:**
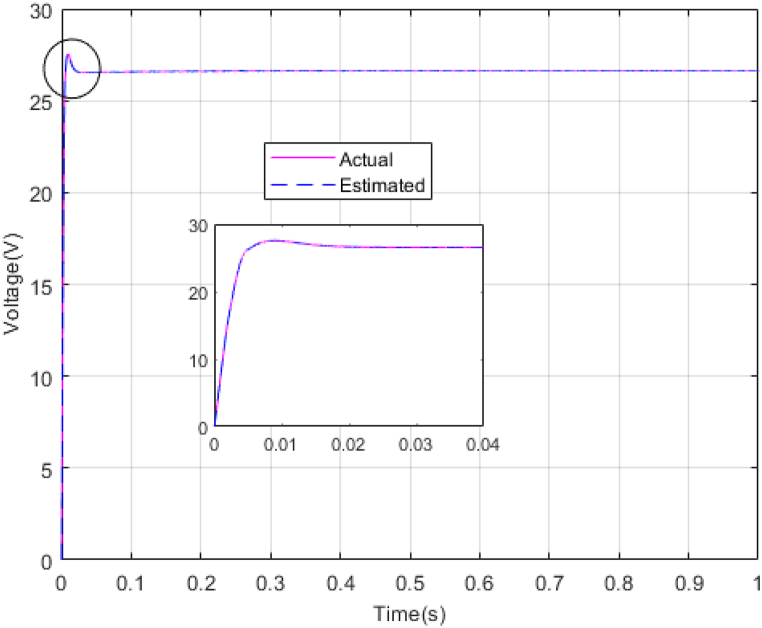
Estimating $V_{pv}$ by NESO

**Fig. 10:**
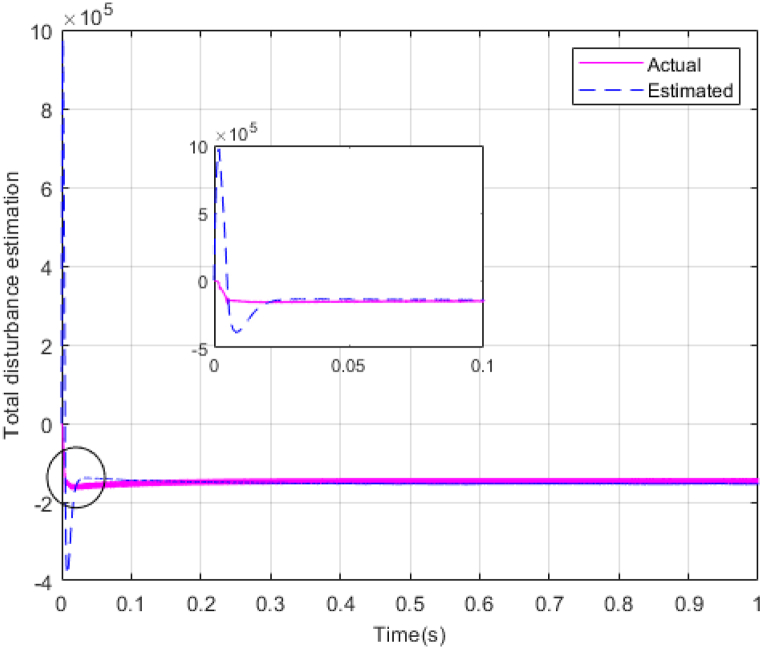
Estimating total disturbance by NESO.

The performance of the ST-ADRC approach is investigated with computer simulations by MATLAB software for the photovoltaic power generation system. A PV panel system is considered, which includes a DC-DC boost converter and 200 W module. PV panel parameters are presented in Table 1. A 10 % deviation is considered on all system parameters and the switching frequency of the converter is set to 50 kHz. The simulations have been done under the conditions of $1000\;W/m^{2}$ irradiance and of 25°C cell temperature. The results are compared with the TSMC controller presented in Ref. [21], SMC controller presented in Ref. [18], and PI controller. The simulation results without external disturbance are presented in Fig. 5, Fig. 6, Fig. 7, Fig. 8, Fig. 9, Fig. 10.

As shown in the comparison diagrams, the ST-ADRC has the best convergence speed and has become stable in about 0.02 s. TSMC and SMC controllers are in the next rank in terms of convergence speed, and the PI controller has the worst performance. This is well illustrated in Fig. 5, Fig. 6. According to Fig. 7 The maximum power point tracking in ST-ADRC is faster than the TSMC method, however, there is no oscillation during tracking in these two methods, while there is some chattering in the PI method. Due to the presence of TD in the ST-ADRC control loop, the system has less overshoot and undershoot, and has a better performance.

λ is very important in adjusting control gains, because; It increases the speed of convergence, but at the same time it increases the duty ratio and the undershoot and overshoot. Also, increasing the gains of $k_{1}$ and $k_{2}$ will improve the performance of the controller, but it will also increase chattering. Therefore, the gains are selected in the most optimal mode so that the output voltage reaches the desired value in the shortest time and the amount of control effort does not exceed its limit. This point is well illustrated in the control effort diagram of Fig. 8.

The ST-ADRC controller is dependent on its extended state observer due to the demand for feedback on errors, which causes the complexity of the proposed method, but on the other hand, it also results in the lack of dependence on the output of the sensors.

**Fig. 11:**
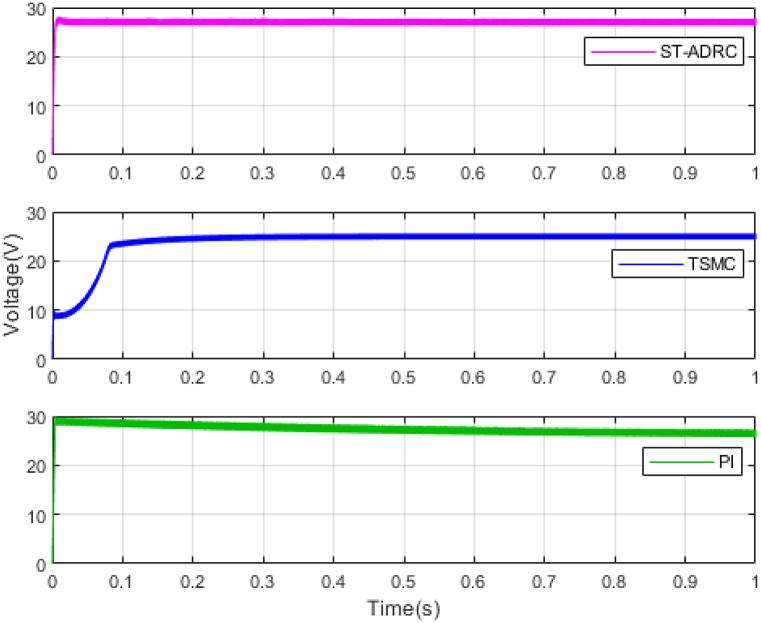
PV panel voltage (V) under disturbance.

The gains of the non-linear extended state observer also have a great impact on the estimation of system states and system uncertainties. The good design of the NESO in Fig. 10, Fig. 11 are proved by voltage estimation as well as total disturbance estimation. This observer has well estimated the system state variables and also the unmodeled dynamics of the system.

A comparison of four control methods in terms of settling time, power losses, maximum error, mean squared error and oscillation at transient in the absence of external disturbance is presented in Table 3.

**Table 3:** Comparison of control methods in the absence of external disturbances.

Controller	Settling Time(s)	Power losses(W)	Maximum Error	Mean Squared Error (MSE)	Oscillation at Transient
ST-ADRC	0.02	0.21	0.0431	0.25	No
TSMC	0.42	0.6	0.0568	0.3	No
SMC	0.47	0.63	0.2362	0.2	Ignorable
PI	0.78	0.7	2.5165	0.61	Ignorable

According to Table 3, the voltage in the ST-ADRC method has reached the desired value faster than the other two methods. After that is TSMC, after that SMC, and at the end is the PI controller, which has the slowest convergence time. The amount of lost power is also better in the proposed method than in other methods. It also performs better in other error calculation methods and the SMC and PI controller has the worst performance. During maximum power point tracking, no fluctuations are observed in both ST-ADRC and TSMC methods, but in the SMC controller and PI controller, a slight fluctuation in voltage and power is observed, which can be ignored. Of course, this value is higher in the PI controller.

**Fig. 12:**
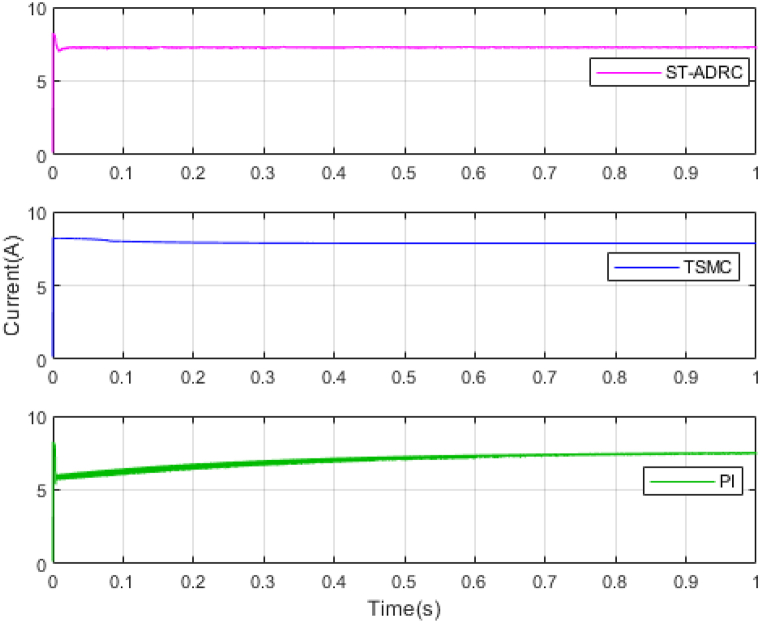
PV panel current (A) under disturbance.

**Fig. 13:**
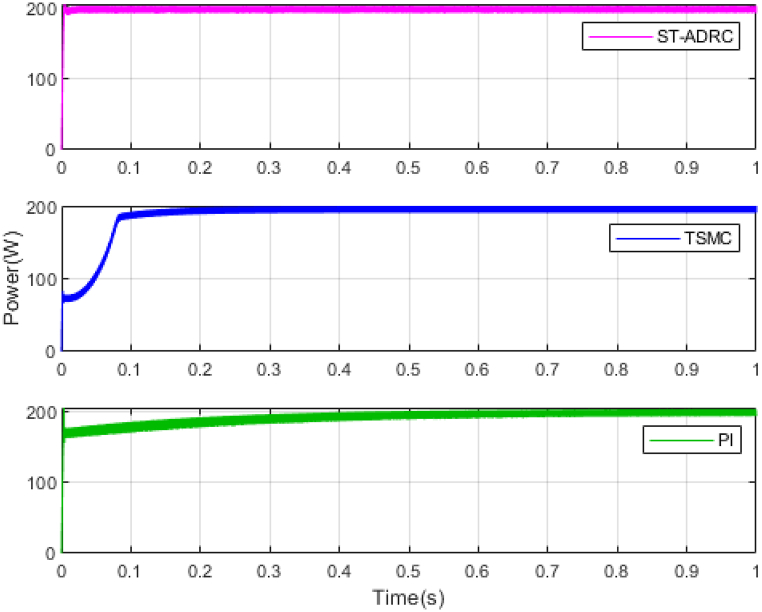
PV panel power (W) under disturbance.

In the next step, two disturbance models are added to system. A sinusoidal disturbance is added to the input voltage of the system as an external disturbance. Its amplitude is 5 V and its frequency is 50 KHz. Also, 15 % uncertainty has been applied to the load resistance as an internal disturbance. The results are presented in Fig. 11, Fig. 12, Fig. 13.

According to Fig. 13 by applying external disturbance and internal disturbance, the system has been able to reach the maximum value of the tracking power. The reason for this can be the inherent ability of sliding mode controls, which are very resistant to disturbances. But again, the ST-ADRC method has a better convergence speed than the control method presented in reference [21]. The PI control method also has the lowest convergence speed and has more chattering to reach the reference path Fig. 11.

**Fig. 14:**
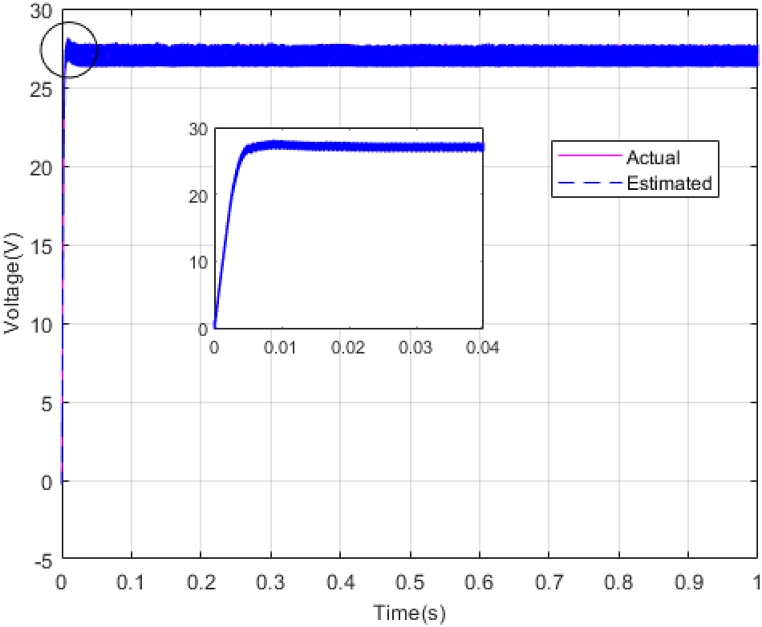
Estimating $V_{pv}$ by NESO

**Fig. 15:**
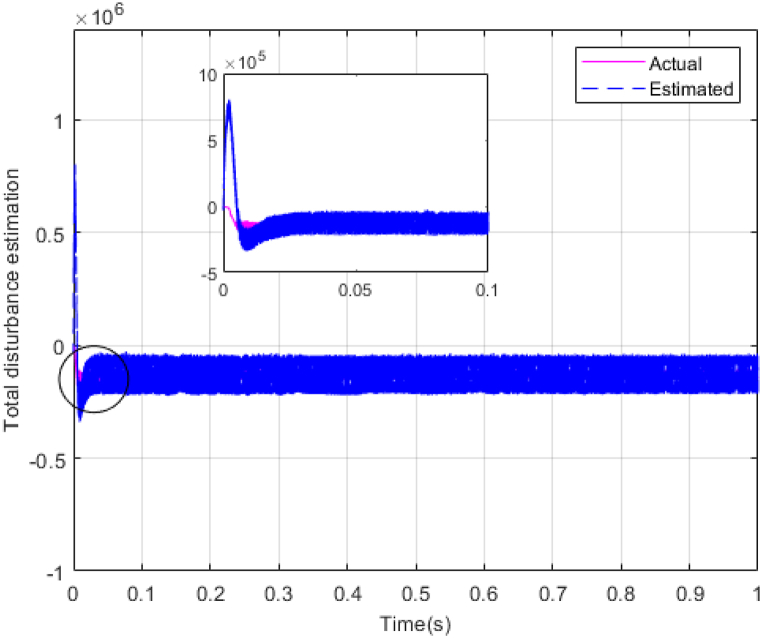
Estimation of total disturbance by NESO.

Fig. 14, Fig. 15 show voltage estimation and total disturbance estimation under sinusoidal external disturbance and uncertainty on resistance. The results are not much different from the results of no disturbance, which indicates the good performance of the proposed method in removing disturbances. The comparison of control methods in the presence of disturbance is presented in Table 4.

**Table 4:** Comparison of control methods under external disturbances.

Controller	Settling Time(s)	Power losses(W)	Maximum Error	Mean Squared Error (MSE)	Oscillation at Transient
ST-ADRC	0.03	0.28	0.7261	0.9832	Ignorable
TSMC	0.5	5.7	0.9892	2.5776	Ignorable
PI	0.88	6.1	5.9412	3.0222	Low

According to Table 4, it is clear that the ST-ADRC controller has less settling time than the other two methods and also loses less power. It performs better in all error calculation methods under external disturbances and internal uncertainties. In methods ST-ADRC and TSMC, there are slight fluctuations in voltage and power, which can be completely ignored. These fluctuations are more in the PI controller.

By comparing with Table 3, it is clear that the error values in the presence of disturbance and the absence of disturbance are not much different in the ST-ADRC controller. The reason for this lack of difference in the ST-ADRC method is the presence of a non-linear extended state observer in the closed loop system, which can estimate the error and compensate for it well. The comparisons prove the better performance and accuracy of the presented control method well in the case of without and with disturbance.

**Fig. 16:**
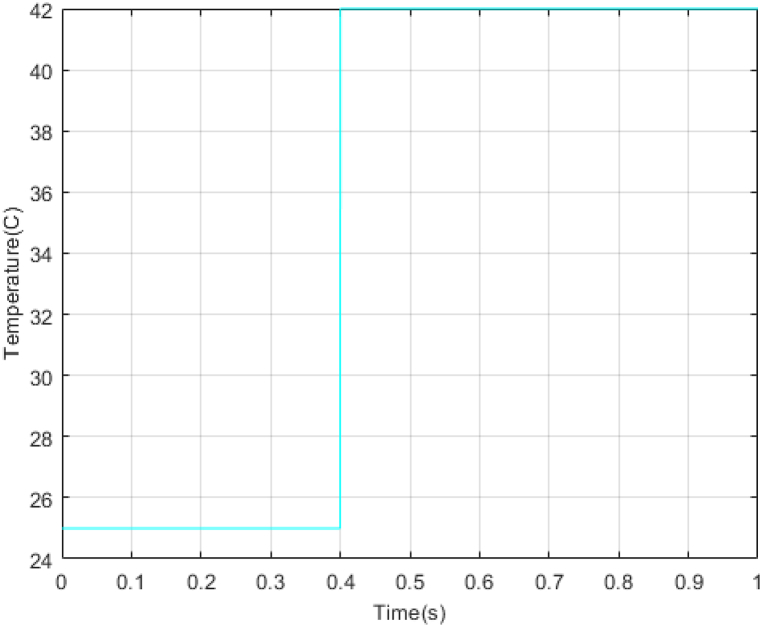
PV panel temperature (C) in new scenario.

**Fig. 17:**
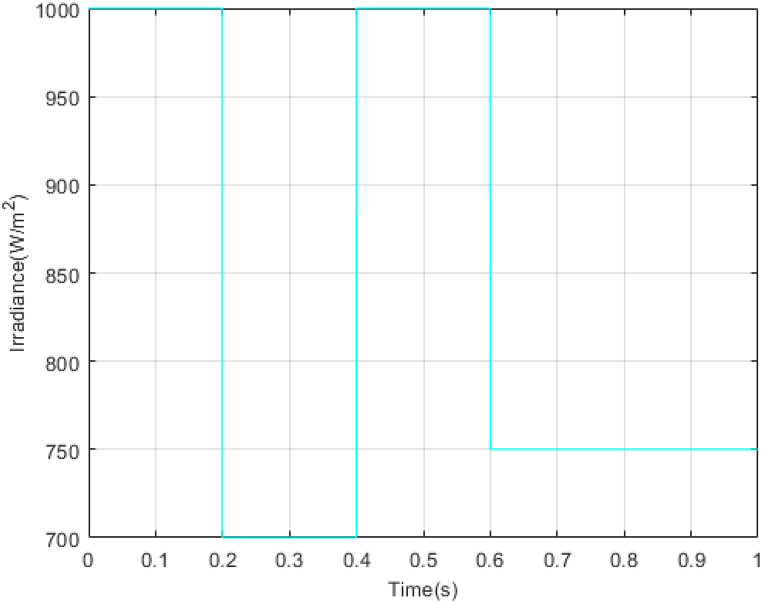
PV panel irradiance $\left(W/m^{2}\right)$ in new scenario.

**Fig. 18:**
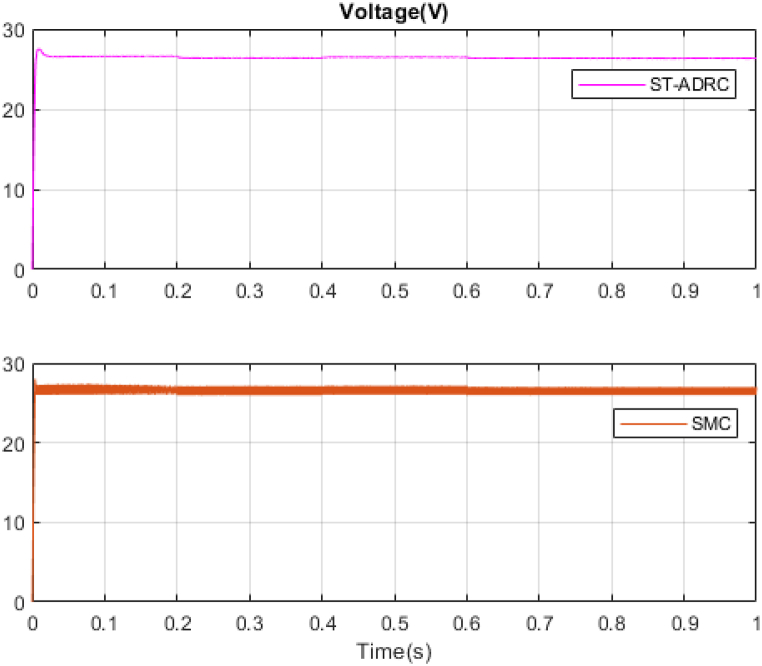
PV panel voltage (V) in new scenario.

**Fig. 19:**
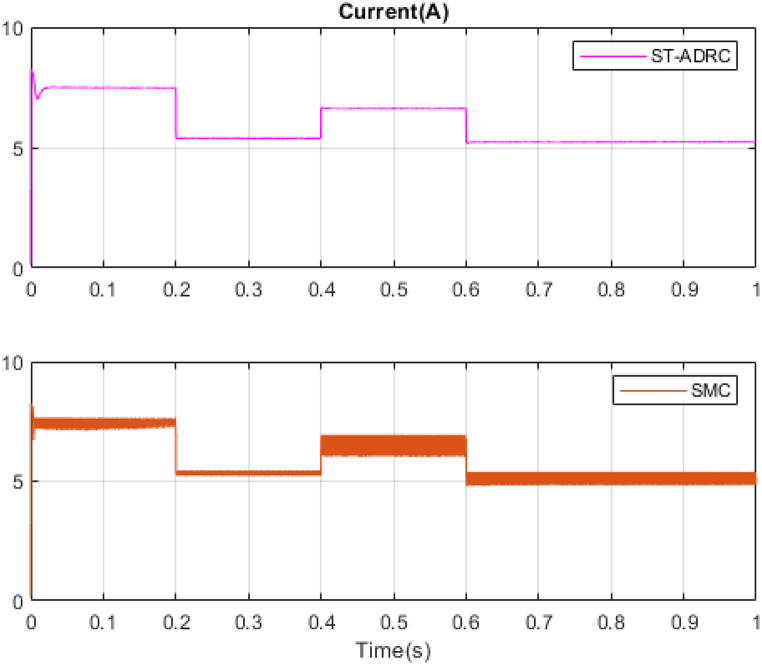
PV panel current (A) in new scenario.

**Fig. 20:**
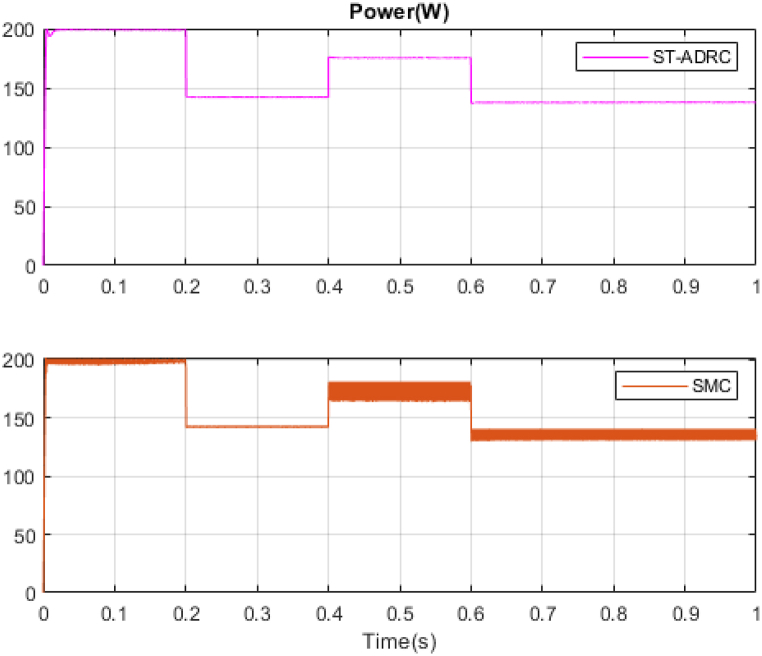
PV panel power (W) in new scenario.

**Fig. 21:**
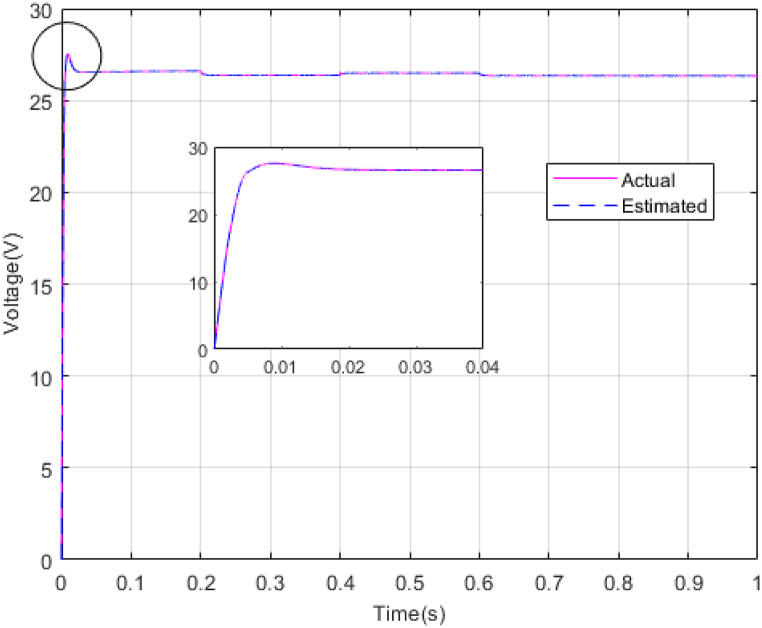
Estimating $V_{pv}$ by NESO in new scenario.

**Fig. 22:**
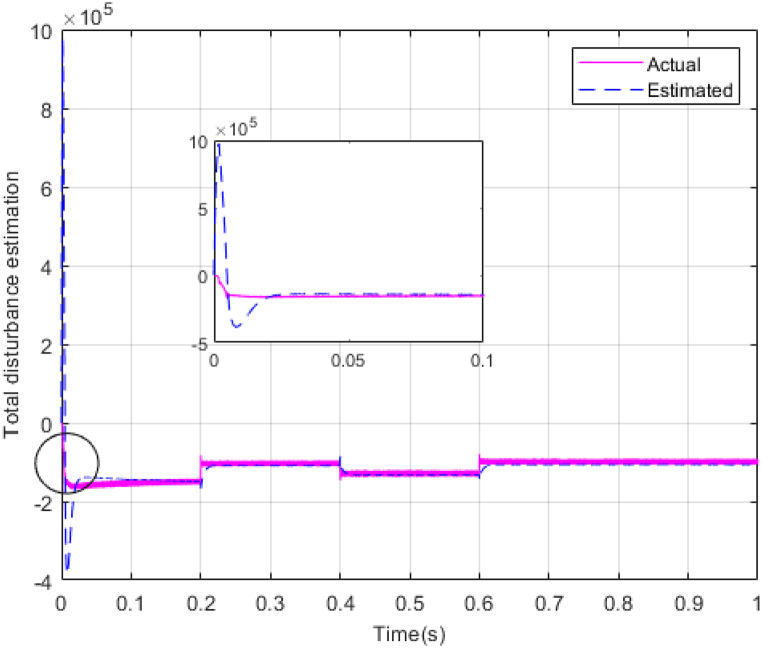
Estimation of total disturbance by NESO in new scenario.

In the next step, a new scenario for radiation and temperature is defined to check the controller's performance under temperature changes and intense radiation. The results of this analysis are compared with the methodology outlined in Ref. [18] and presented in Fig. 16, Fig. 17, Fig. 18, Fig. 19, Fig. 20, Fig. 21, Fig. 22.

In Fig. 16, Fig. 17, temperature and radiation are shown in the new scenario. Based on the data presented in Fig. 18, Fig. 19, Fig. 20, the STA-ADRC controller demonstrates superior performance in maintaining the desired voltage and power levels despite fluctuations in the system's radiation and temperature. Notably, the proposed controller exhibits minimal oscillation at peak points, in contrast to the classical controller, which displays significant chattering that intensifies at these critical junctures.

Fig. 21, Fig. 22 illustrate the voltage estimation and disturbance observation for the entire system. The observer demonstrates high accuracy in estimating system dynamics, effectively tracking changes. This robust estimation capability is crucial for the overall system performance and stability. The performance and resistance of the proposed method are also proven under different temperatures and radiation. The comparison of control methods under variations in radiation and temperatures is presented in Table 5.

**Table 5:** Comparison of control methods under variations in radiation and temperatures.

		ST-ADRC		SMC	
States	$P_{MPP}$	$P_{PV}$	$\frac{P_{pv}}{P_{MPP}}*100\%$	$P_{PV}$	$\frac{P_{pv}}{P_{MPP}}*100\%$
State 1: $1000W/m^{2},25{}^{\circ}C$	200W	199.8W	99.89%	199.3W	99.68%
State 2: $700W/m^{2},25{}^{\circ}C$	142W	141.9W	99.9%	139.2W	98.02%
State 3: $1000W/m^{2},42{}^{\circ}C$	188W	175.8W	93.51%	172.4W	91.70%
State 4: $750W/m^{2},42{}^{\circ}C$	143W	137.8W	96.36%	135.1W	94.47%

Analysis of Table 5 demonstrates that the proposed controller exhibits superior performance across all operational modes compared to the classical sliding mode controller. The quantitative comparison suggests that the proposed controller offers a marked improvement in both precision and energy conservation, which are critical factors in optimizing system performance and sustainability. The proposed system has been subjected to analysis under four distinct conditions. The system's responses to variations in irradiance and temperature were observed and recorded. The accuracy of the Maximum Power Point Tracking (MPPT) algorithm in identifying the Maximum Power Point (MPP) ranged from 93.51 % to 99.9 %. The targeted efficiency of the system has been achieved to a significant degree.

### 5.1 Real-time simulation

Owing to the escalating intricacy and financial expenditures associated with projects, coupled with the mounting urgency to curtail the duration required for market introduction, the processes of evaluating and substantiating the functionality of intricate systems have assumed heightened significance within the design phase.

Over the past two decades, commercially available computers have become increasingly powerful and affordable. This has led to the emergence of highly sophisticated simulation software applications capable of high-fidelity dynamic system simulations and associated control systems, as well as automatic code generation for implementation in industrial controllers [45]. Due to the advancement of software tools like MATLAB/Simulink with its Real-Time Workshop (RTW) and Real-Time Windows Target (RTWT), real-time simulators are extensively used in many engineering fields, including industry, education, and research institutions.

In this study, to further investigate the proposed method rigorously, the non-real-time simulation results have been compared with real-time simulation results. The simulations have been done in MATLAB/Simulink environment and under the uncertainties. The results are presented in Fig. 16, Fig. 17, Fig. 18, Fig. 19, Fig. 20, Fig. 21.

**Fig. 23:**
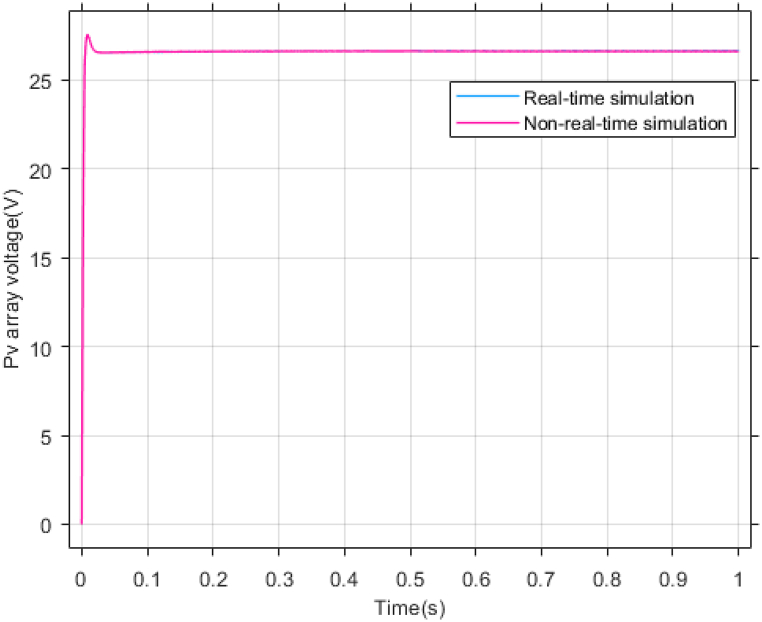
PV panel voltage (V).

**Fig. 24:**
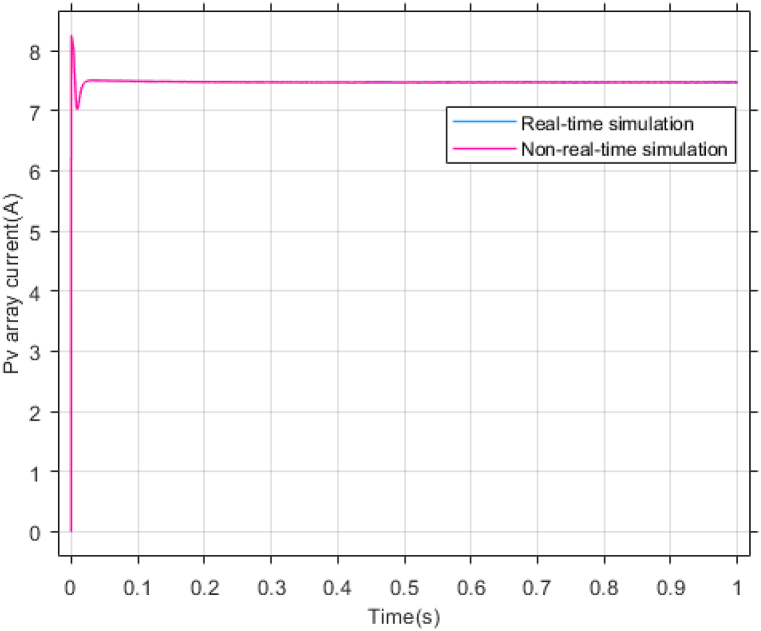
PV panel current (A).

**Fig. 25:**
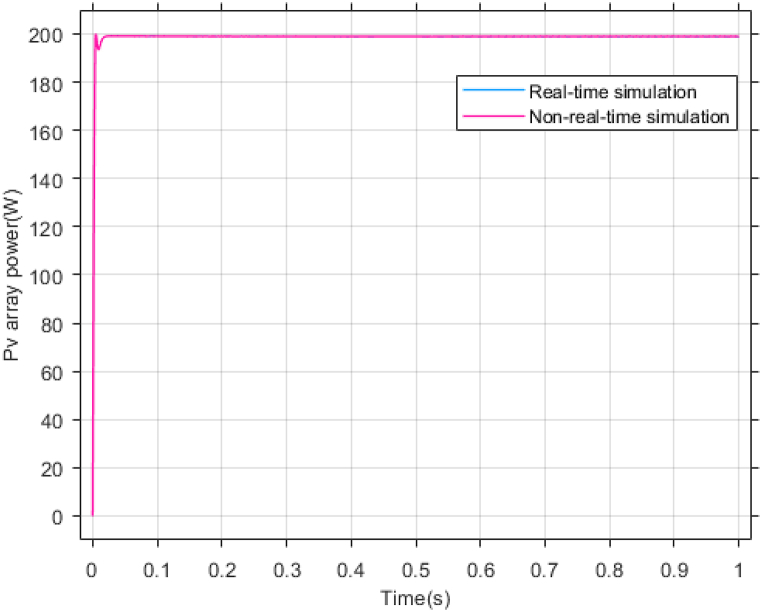
PV panel power (W).

**Fig. 26:**
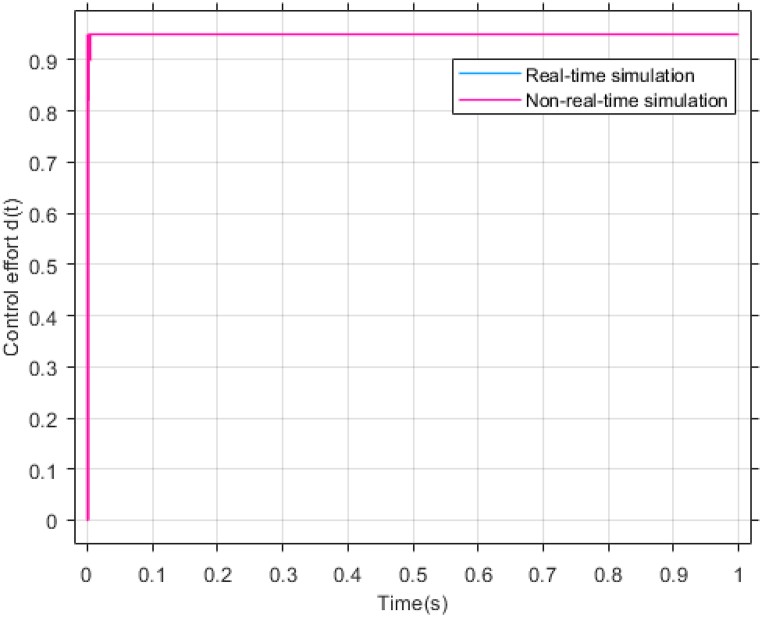
PV Control effort d(t).

According to Fig. 23, Fig. 24, Fig. 25, the simulation results of non-real-time and real-time have very little difference and have reached stability almost at the same time. Fig. 26 shows the lack of control effort difference in each simulation.

**Fig. 27:**
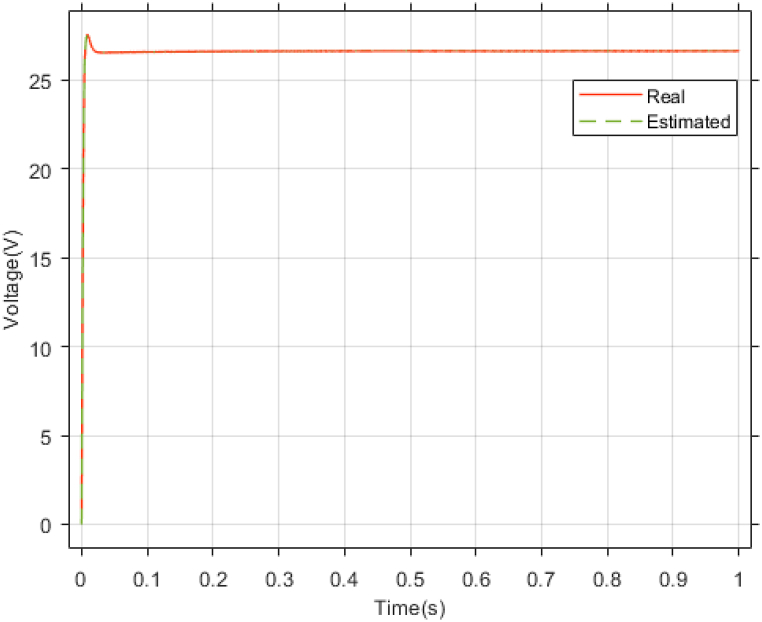
Estimating $V_{pv}$ by NESO in real-time simulation.

**Fig. 28:**
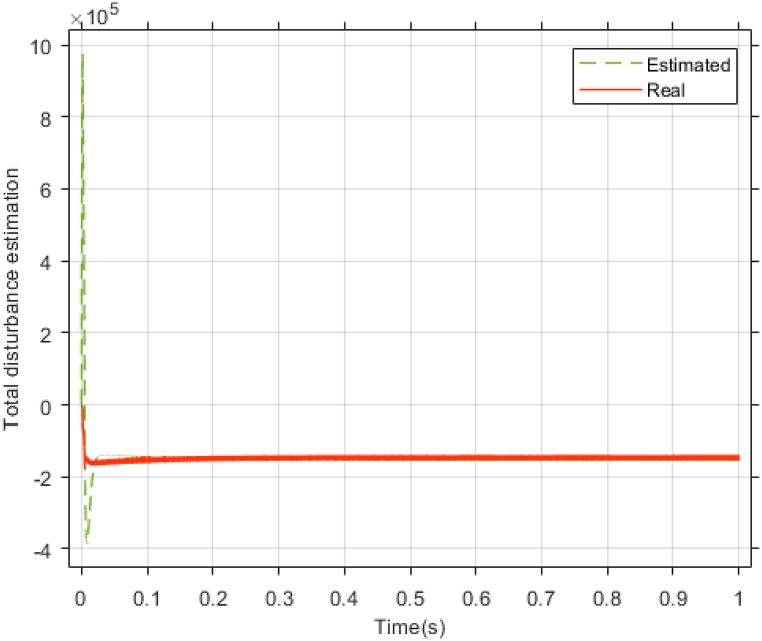
Estimation of total disturbance by NESO in real-time simulation.

Fig. 27, Fig. 28 presents voltage estimation and total disturbance estimation in real-time simulation and show the proper performance of the non-linear extended state observer in voltage and total disturbance estimation in real-time.

By conducting comprehensive simulations and statistical analysis, the efficacy and robustness of the proposed approach were thoroughly assessed and validated against real-time simulations and benchmark methods.

This high level of accuracy in non-real-time modeling and simulation has led to a minimal difference between the results obtained from non-real-time simulations and real-time simulations. This affirms the validity of the proposed method and the presented results in the manuscript, demonstrating the proposed method's suitability for practical implementation in real photovoltaic systems.

Moreover, the close agreement between the simulation results and real-world performance indicates the robustness and reliability of the proposed approach. By accurately capturing the system dynamics and environmental conditions in the simulations, the author has ensured that the proposed method can effectively translate its simulated performance to real-world scenarios, further reinforcing the practical applicability of the presented work.

## 6 Conclusion

In this paper, active disturbance rejection control based on a super-twisting sliding mode is proposed for the maximum power point tracking of PV power generation systems. First, the PV panel model and boost converter are introduced. Next, the strong coupling between channels, model uncertainties, and low oscillation frequency are considered total disturbances and are estimated by a non-linear extended state observer.

In the next step, the ST-ADRC control is applied to track the maximum power, and the estimated total disturbance in the controller is compensated online. To reduce overshoot and reduce noise, an optimal tracking differentiator has been used, the output of which is a clean differential signal without noise, and it also increases the speed of convergence. The closed-loop stability of the non-linear extended state observer and controller is proved. The results show that the proposed method can significantly improve the response speed of the system and reach the maximum power, reducing the fluctuation range. The performance of the controller and observer under extreme changes in temperature and radiation, external disturbances, and internal uncertainties have been compared with other applied methods such as PI controller, SMC, and TSMC. The results confirm the effectiveness and superiority of the proposed method under disturbance and no disturbance.

Therefore, it can be concluded that the control method presented in this article is an effective method with high and robust tracking accuracy.

Considering the high dependence of the control method on its gains, it is better to aim for future works to improve the method of adjusting the control gains using various optimization methods including reinforcement learning, neural networks, etc.

## CRediT authorship contribution statement

**Amir Hossein Raouf:** Writing – original draft, Investigation, Data curation. **Fatemeh Sadat Yazdiniya:** Writing – review & editing, Software, Methodology, Investigation, Formal analysis, Data curation. **Gholam Reza Ansarifar:** Writing – review & editing, Supervision, Methodology, Conceptualization.

## Declaration of competing interest

The authors declare that they have no known competing financial interests or personal relationships that could have appeared to influence the work reported in this paper.
